# Unusual presentation of secondary syphilis mimicking erythema multiforme in HIV positive patient: a case report

**DOI:** 10.11604/pamj.2023.46.55.41497

**Published:** 2023-10-16

**Authors:** Amira Suryani Rahmatika, Dwi Murtiastutik, Afif Nurul Hidayati, Astindari, Maylita Sari, Septiana Widyantari, Regitta Indira Agusni, Linda Astari

**Affiliations:** 1 Department of Dermatology and Venereology, Faculty of Medicine, Universitas Airlangga, Dr. Soetomo General Academic Hospital, Surabaya, Indonesia

**Keywords:** Secondary syphilis, erythema multiforme, targetoid lesions, HIV, case report

## Abstract

Secondary syphilis is known as “The Great Imitator”. It can mimic numerous diseases clinically and histologically, including erythema multiforme (EM). Coinfection with HIV often makes its manifestations more atypical leading to delays in diagnosis and therapy. A 34-year-old male-sex-male patient who had received coronavirus disease 2019 (COVID-19) vaccine 1 week earlier presented with complaints of slightly pruritic scaly erythematous targetoid plaques and erythematous macules on the trunk and extremities for 6 weeks. Histopathology examination showed basal cell vacuolar degeneration of the epidermis and lymphocytic infiltrates along the dermal-epidermal junction and superficial dermis, consistent with EM. Upon further investigation, syphilis and HIV serology were reactive (VDRL 1: 128, TPHA 1: 40960, CD4+ 461 cells/µl). Lesions improved significantly after a single dose of 2,4-million units of benzathine penicillin intramuscular injection. Secondary syphilis presenting as erythema multiforme (EM)-like eruptions is very rare. Physicians should be aware of this unusual presentation to prevent complications.

## Introduction

Syphilis prevalence is still high, especially among the key populations in the world, such as the male-sex-male group. The key population also becomes an important cofactor of human immunodeficiency virus (HIV) transmission [1]. Cutaneous manifestations presenting as EM-like eruption in secondary syphilis are very uncommon, thus making the diagnosis challenging. Furthermore, HIV coinfection often makes syphilis manifestations more atypical [2]. We describe an atypical case of a 34-year-old male-sex-male patient with secondary syphilis and HIV coinfection resembling erythema multiforme clinically and histologically who showed significant improvement with a single dose of benzathine penicillin injection of 2,4 million IU therapy.

## Patient and observation

**Patient information:** a 34-year-old male-sex-male patient with a history of receiving 1^st^ dose of coronavirus disease 2019 (COVID-19) vaccine 1 week earlier presented with complaints of slightly pruritic red skin patches on his trunk and extremities, including his palms and soles, for 6 weeks. No history of fever, cough, running nose, sore throat, mouth ulcers, weight loss, hair loss, genital ulcers, or mouth ulcers. There was also no history of taking medicines and food supplements, smoking, drinking alcohol, and a blood transfusion before. No history of the same complaints in his family.

**Figure 1:**
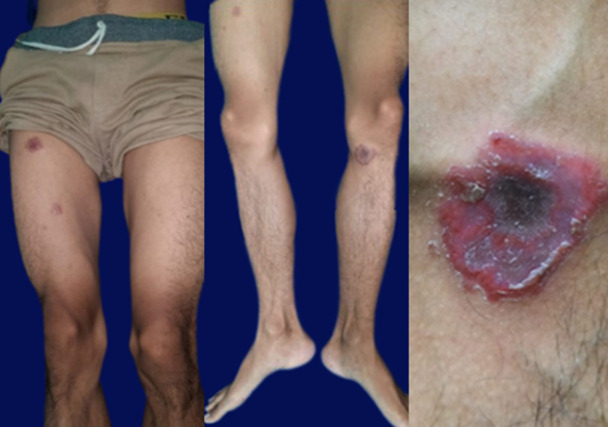
targetoid lesions on the lower extremities

**Figure 2:**
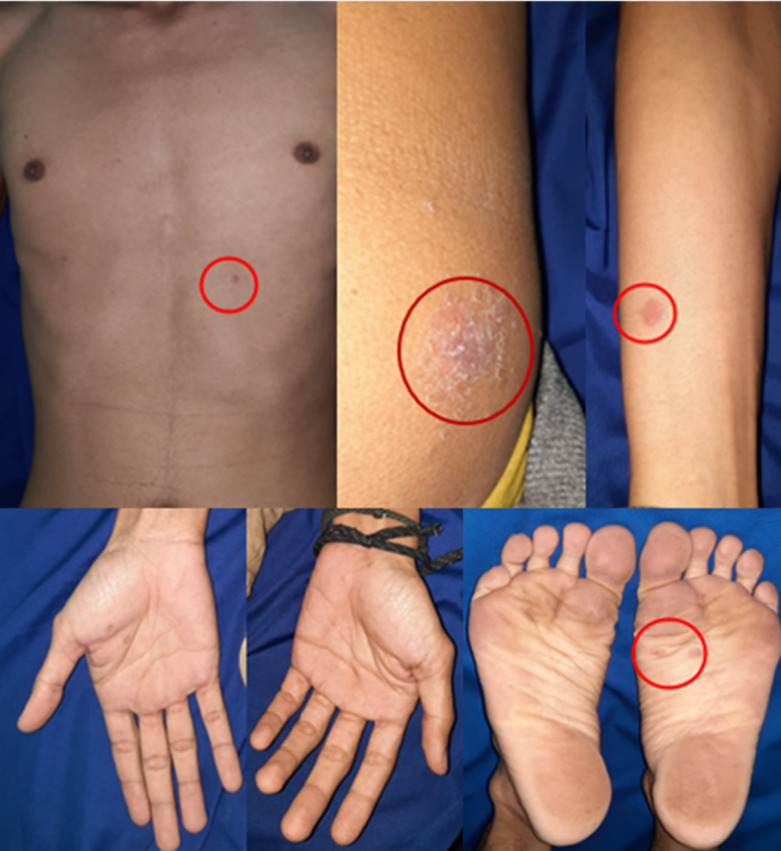
the clinical manifestations on the trunk and extremities

**Clinical Findings:** the patient was well-oriented and had normal vital signs. The physical examination showed multiple sharply demarcated erythematous annular scaly plaques with a necrotic area in the center which looked like targetoid lesions with a diameter between 3-6 cm on his lower extremities (Figure 1). There were also multiple erythematous macules and plaques on his trunk and upper extremities, some lesions were scaly and confluences. Palms and soles are also affected (Figure 2). No lymphadenopathy or lesions were found.

**Figure 3:**
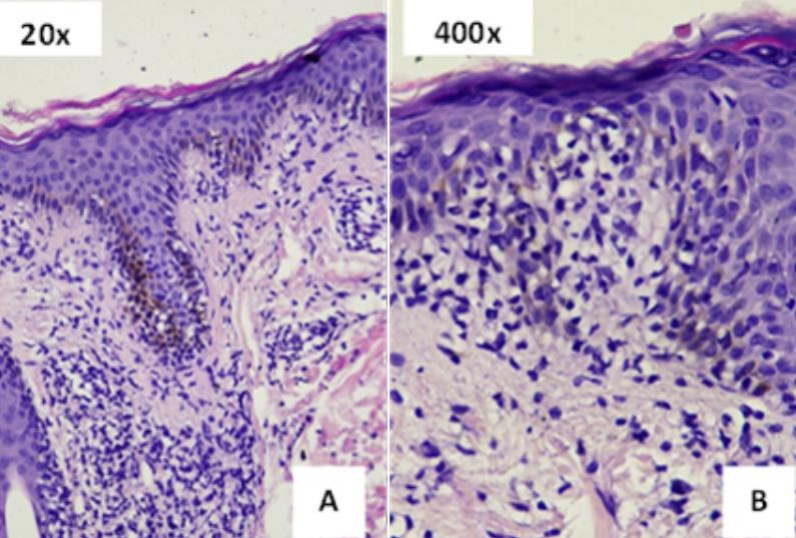
the histopathology examination; A) the histopathology examination revealed the epidermis with basal cell vacuolar degeneration and the dermis layer showed a lymphocytic infiltrate along the dermal-epidermal junction and the superficial dermis layer which was compatible with erythema multiforme (20x magnification); B) histopathology of the same lesion with 400x magnification

**Diagnostic assessment:** the potassium hydroxide examination was negative. The IgM and IgG anti-herpes simplex virus (HSV)-1 and anti-HSV-2 serology examinations were non-reactive. The histopathology examination revealed basal cell vacuolar degeneration in the epidermal layer and the dermal layer showed lymphocytic infiltrates along the dermal-epidermal junction and the superficial dermis, consistent with EM (Figure 3). The dark field microscope examination showed negative results for spirochetes. The blood test results were reactive for venereal disease research laboratory (VDRL) with a titer of 1: 128 and *Treponema pallidum* hemagglutination assay (TPHA) with a titer of 1: 40960, while the HIV serology was reactive with a cluster of differentiation-4 (CD4)+ value of 461 cells/µl. Complete blood count examination showed normal Hb 13,5 g/dl, thrombocytes 46.000/µl, white blood cells 8.500/µl, eosinophil 3%, basophil 1%, neutrophil 69%, monocytes 11%, lymphocytes 16%. Immunohistochemistry and the Warthin-Starry technique for finding spirochetes in tissue sections of secondary syphilis lesions couldn´t be performed due to unavailability in our hospital. Polymerase chain reaction (PCR) examination for treponema also could not be done due to unavailability.

**Diagnosis:** secondary syphilis with HIV-coinfection.

**Therapeutic interventions:** the patient received 2,4 million IU of single-dose benzathine penicillin along with antiretroviral therapy.

**Follow-up and outcome of interventions**: the lesions showed significant improvement with a decline of syphilis serological titer after 3 months follow-up became VDRL 1: 4 and TPHA 1: 640, and after 6 months follow-up became VDRL 1: 1 and TPHA 1: 320.

**Patient perspective:** “I was shocked when I found out that I got syphilis and HIV positive, but I feel more relieved because I can get the proper treatment and some of the red patches have disappeared. Hopefully, I can get treatment more regularly and my condition will be improved.”

**Informed consent:** it has been signed by the patient.

## Discussion

The presence of erythematous plaques resembling a target lesion with a necrotic area in the center and a history of coronavirus disease 2019 (COVID-19) vaccination, in this case, led to suspicion of erythema multiforme (EM) due to vaccination as the initial assessment after eliminating herpes simplex virus (HSV) infection and medications as the common trigger factors of EM. Secondary syphilis is suspected as a differential due to the history of being MSM and the presence of erythematous lesions on the palms and soles which are typical for secondary syphilis lesions. Syphilis diagnosis needs a combination of a thorough medical history (including sexual history), clinical symptoms, and additional examinations. Two laboratory serologic tests are necessary for a presumptive diagnosis of syphilis: a nontreponemal test, such as the Venereal Disease Research Laboratory (VDRL), and a treponemal test, such as the *Treponema pallidum* hemagglutination assay (TPHA). Examination using a dark field microscope as the definitive method can detect *T. pallidum* directly from exudates or tissue, but it is usually limited because of unavailability in each healthcare facility and the lesions on keratinized skin (palmoplantar lesions and maculopapular rash on the body) typically do not contain enough treponemes to give a positive result [1,2].

Erythema multiforme (EM) is a rare, immune-mediated disease of cutaneous or mucocutaneous eruption characterized by “target” lesions that typically affect the face and extremities, but can also affect palms and soles and induce burning or itching symptoms [3,4]. The course of EM is often acute, mild, and self-limited, but carries a risk of relapse and may be persistent in some cases. The precipitating event relates to infection in 90% of cases, while less than 10% are caused by medications. The most commonly identified etiology is HSV type 1, while it has also been linked to HSV-2 and other infections. Vaccines have also been linked to EM, which is probably an epiphenomenon of immunogenicity to viral antigens, but the incidence is low [5]. The majority of erythema multiforme cases don't need any additional diagnostic procedures, however, histopathological analysis may be helpful to confirm the diagnosis as well as to differentiate between EM and other diagnoses [5,6]. Histopathology examination of EM exhibits a significant dermal inflammatory infiltration, liquefactive necrosis, degeneration, and dyskeratotic keratinocytes in the epidermis that lies above. The dermo-epidermal interface is obscured by a lichenoid reaction pattern, which is characterized by a mild to moderate infiltrate of lymphocytes, some of which migrate into the basal layer. Additionally, there is some epidermal spongiosis and basal vacuolar alteration [3]. The location of the skin biopsy, as well as the time point of the biopsy during the disease course, affect the histological characteristics of EM [6].

Meanwhile, the histological pattern of secondary syphilis varies greatly, with neutrophils in the stratum corneum, irregular/psoriasiform acanthosis, effacement of the rete ridges/elongated rete ridges, vacuolar interface with vacuolar predominance/with equal numbers of lymphocytes and vacuoles, endothelial swelling, presence of plasma cells, lymphocytes with ample cytoplasm, and interstitial inflammation [7]. Although most of the studies found that plasma cells are the most common finding in secondary syphilis, up to one-third of all biopsies may have scant or absent plasma cells, and the vascular changes may not be prominent. The histopathologic examination for secondary syphilis has low specificity and low sensitivity since the features can also be seen in conditions clinically mimicking secondary syphilis maculopapular lesions [7,8]. The Warthin-Starry technique or the Steiner variant of the Dieterle technique are the two most used methods for finding spirochetes in tissue sections of secondary syphilis lesions. The organisms were primarily found near the dermal-epidermal junction [9]. Immunohistochemistry is superior for detecting spirochetes compared with silver stains, however, it may not always be routinely available at many healthcare facilities. There are still certain cases where both immunohistochemistry and silver stains are unable to detect organisms [7].

The histological finding, in this case, revealed basal cell vacuolar degeneration of the epidermis and lymphocytic infiltrates along the dermal-epidermal junction and superficial dermis. It did not show any plasma cells and spirochetes. Silver stain or immunohistochemistry examination could not be performed due to unavailability in the hospital. Secondary syphilis diagnosis was then made based on history taking, clinical manifestations, and syphilis serologic results. The patient showed rapid clinical resolution after a single dose of benzathine penicillin 2,4-million-unit intramuscular injection. Patient achieved serological cure by the decline of VDRL titer more than four-fold to 1: 4 after 3 months of follow-up and 1: 1 after 6 months of follow-up. It is important to raise suspicion of secondary syphilis in patients with sexual risk behavior. The key to early identification and treatment of secondary syphilis is the combination of clinical pathologic correlation and serologic testing. Accurate diagnosis can lead to proper treatment and prevent complications.

## Conclusion

This case highlights that the dermatological manifestations of secondary syphilis are highly variable, earning it the name of “The Great Imitator”. Erythema multiforme (EM)-like lesions are an uncommon presenting sign of secondary syphilis. Coinfection with HIV may play a part in the atypical presentation of the lesions. Even though rare in occurrence, syphilis should be considered in the differential diagnosis of HIV patients presenting with targetoid lesions. A single dose of benzathine penicillin 2.4-million-unit intramuscular injection is sufficient to achieve a serological cure for early syphilis with HIV coinfection like in this case as indicated by a decrease in the nontreponemal titer more than 4-fold after 6 months of follow-up. Accurate diagnosis will lead to appropriate management of the patient.
